# Exploring the anticancer activities of Sulfur and magnesium oxide through integration of deep learning and fuzzy rough set analyses based on the features of Vidarabine alkaloid

**DOI:** 10.1038/s41598-024-82483-8

**Published:** 2025-01-17

**Authors:** Heba Askr, Marwa A. A. Fayed, Heba Mamdouh Farghaly, Mamdouh M. Gomaa, Enas Elgeldawi, Yaseen A. M. M. Elshaier, Ashraf Darwish, Aboul Ella Hassanien

**Affiliations:** 1 Faculty of Computers and Artificial Intelligence, University of Sadat City, Sadat City, Egypt; 2 Department of Pharmacognosy, Faculty of Pharmacy, University of Sadat City, Sadat City, 32897 Egypt; 3 Computer Science Department, Faculty of Science, Minia University, Minya, Egypt; 4 Department of Organic and Medicinal Chemistry, Faculty of Pharmacy, University of Sadat City, Sadat City, 32897 Menoufia Egypt; 5 Faculty of Science, Helwan University, Cairo, Egypt; 6 Faculty of Computer and AI, Cairo University, Giza, Egypt; 7 Scientific Research School of Egypt (SRSEG), https://egyptscience-srge.com

**Keywords:** Drugs repurpose, Drug discovery and development, Marine natural products, Virtual screening, Explainable Artificial Intelligence (XAI), Deep learning (DL), Fuzzy Rough Set (FRS), Cytotoxicity, Computational biology and bioinformatics, Drug discovery

## Abstract

Drug discovery and development is a challenging and time-consuming process. Laboratory experiments conducted on Vidarabine showed IC_50_ 6.97 µg∕mL, 25.78 µg∕mL, and ˃ 100 µg∕mL against non-small Lung cancer (A-549), Human Melanoma (A-375), and Human epidermoid Skin carcinoma (skin/epidermis) (A-431) respectively. To address these challenges, this paper presents an Artificial Intelligence (AI) model that combines the capabilities of Deep Learning (DL) to identify potential new drug candidates, Fuzzy Rough Set (FRS) theory to determine the most important chemical compound features, Explainable Artificial Intelligence (XAI) to explain the features’ importance in the last layer, and medicinal chemistry to rediscover anticancer drugs based on natural products like Vidarabine. The proposed model aims to identify potential new drug candidates. By analyzing the results from laboratory experiments on Vidarabine, the model identifies Sulfur and magnesium oxide (MgO) as new potential anticancer agents. The proposed model selected Sulfur and MgO based on Interpreting their promising features, and further laboratory experiments were conducted to validate the model’s predictions. The results demonstrated that, while Vidarabine was inactive against the A-431 cell line (IC_50_ ˃ 100 µg∕mL), Sulfur and MgO exhibited significant anticancer activity (IC_50_ 4.55 and 17.29 µg/ml respectively). Sulfur displayed strong activity against A-549 and A-375 cell lines (IC_50_ 3.06 and 1.86 µg/ml respectively) better than Vidarabine (IC_50_ 6.97 and 25.78 µg/ml respectively). However, MgO showed weaker activity against these two cell lines. This paper emphasizes the importance of uncovering hidden chemical features that may not be discernible without the assistance of AI. This highlights the ability of AI to discover novel compounds with therapeutic potential, which can significantly impact the field of drug discovery. The promising anticancer activity exhibited by Sulfur and MgO warrants further preclinical studies.

**Supplementary Information:**

The online version contains supplementary material available at 10.1038/s41598-024-82483-8.

## Introduction

Cancer is one of the most serious healthcare crises affecting humanity, as well as one of the most difficult tasks for scientists working on novel treatments or developing old ones with fewer adverse effects^1^. Cancer has many challenges in discovering a safe and affordable cost of new treatment and excessive cost of investment in cancer care.

Drug repurposing is the development of new drugs for previously identified medications. Since the creation of new pharmaceuticals is a lengthy and cost-effective procedure, this is one of the emerging trends/options for combating challenging diseases with previously existing treatments^2^. In modern drug discovery, especially in virtual screening (VS) as a main tool of drug repositioning (repurposing), finding screening hits, and optimizing those hits to improve oral bioavailability, metabolic stability, efficacy/potency, affinity, and selectivity will utilize the features of lead drug. However, traditional methods of feature selection which depend on expertise selection are fixed and hand-crafted features and obviously will miss some of the unknown and hidden patterns. To overcome these limitations, engineered craft by implementation of Artificial Intelligence (AI) tools is more accurate, faster, and affordable cost.

Vidarabine is a natural drug isolated from marine sources. It is an FDA-approved antiviral drug^3,4^. This compound has been clinically used in the management of various herpes infections, including encephalitis that can arise in the initial phases^5^. It has been considered the first medicine to be widely accessible in the United States for the parenteral treatment of life-threatening or severe *Herpes simplex* virus infections in humans^6^.

Despite its shown efficacy as a treatment drug against a wide range of viruses, vidarabine has several substantial drawbacks. It is easily metabolized by adenosine deaminase (ADA) to arabino-furanosyl hypoxanthine (Ara-H), which is 10-fold less effective and has poor lipophilicity, resulting in reduced intestinal membrane permeability^7,8^. Furthermore, vidarabine displayed limited pharmacokinetic and pharmacodynamic profiles^9^. Vidarabine was withdrawn from the market as an antiviral ointment due to its toxicity, however, it possesses anticancer effects also. This promising activity of vidarabine guided us to consider it in “lead optimization of drug discovery”^10^. Drug discovery involves many strategies such as virtual screening, high-throughput screening, phenotypic screening, structure-based drug design, fragment-based drug design, and ligand-based drug design^11^. Here we used virtual screening and ligand-based approaches based on vidarabine structure as a lead compound. These two approaches usually utilize public databases for drugs.

AI has found its way into numerous critical real-world applications, including computer networking^12,13^, feature selection^14,15^, agriculture^16^, healthcare^17,18^ and drug discovery^19^. Biomedical data is being utilized to advance drug discovery through AI techniques, uncovering concealed patterns and evidence. Machine learning (ML) and DL methods are employed in drug discovery and development. Consequently, the utilization of AI has become imperative to expedite the drug development process, leading to the identification of new drug candidates, cost savings, exploration of drug repurposing opportunities, and the potential revival of withdrawn or unsuccessful drugs. With the concept of XAI, the goal is to discard the “black box” mentality and towards the “glass box” strategy where all the parameters are known, and there is a clear understanding of how the model arrives at its conclusions, providing complete transparency. Ideally, the model would be fully transparent, but in many cases in DL models, there is a certain degree of explainability, which can be likened to the “glass box” with an opacity degree between 0% and 100%. When the translucent glass box has low opacity (or when the model has high transparency), it promotes a better comprehension of the model^20^.

In drug discovery, XAI has the capability to enhance human intuition and expertise in the creation of new bioactive compounds that possess specific properties. It also promotes collaboration among medicinal chemists, chemo informaticians, and data scientists, facilitating their joint efforts. XAI plays a crucial role in analyzing and interpreting intricate chemical data, enabling the formulation of innovative pharmacological hypotheses. Importantly, XAI helps mitigate the influence of human bias in the drug discovery process, ensuring more objective and reliable outcomes^21^.

The Fuzzy Rough Set (FRS) theory has a substantial impact on the drug discovery industry by providing a robust framework for managing and analyzing data that is uncertain and imprecise. This type of data is commonly encountered in drug development due to the intricate nature of biological systems and the limitations of experimental information. FRS theory allows for the effective handling of vague and uncertain details pertaining to drug molecules, target interactions, and biological activities. By incorporating fuzzy logic, it enables the modeling of knowledge that is ambiguous or incomplete, thus capturing the inherent uncertainties present in drug discovery datasets. In the context of drug discovery, FRS can be employed for feature selection, facilitating the identification of pertinent features or descriptors that contribute to predicting drug activity. This capability is particularly valuable when working with complex molecular datasets that possess a high number of dimensions^22^.

The motivation of this paper is to rediscover a simple and cheap anticancer drug-based VS on the chemical features of Vidarabine utilizing the power of AI and FRS. The departure point commences with the determination of the anticancer and cytotoxicity of vidarabine. While AI is being used to enhance human intuition and expertise in the creation of new bioactive compounds, FRS prioritizes the best compounds from the selected databases. From this DRY LAB phase, the selected compounds were examined against the same cancer cell lines.

Drug discovery is known to be an exceedingly lengthy process. Employing AI methods to enhance the process of drug repurposing through rapid VS has the potential to enhance and expedite the identification of promising drug candidates targeting both communicable and non-communicable diseases. However, very few research using AI in the drug repurposing process has been conducted^23^ .

A review by Selvaraj et al.^24^ has been made to discuss different algorithms and tools for predicting the target in repurposing a drug. They conduct their research based on various sources including PubMed, Google Scholar, and Scopus. Tsou et al.^25^ applied DL in virtual screening. They conducted a comparison study between deep neural networks (DNN) and other ligand-based virtual screening (LBVS) methods. Another review by Quazi^26^ has been conducted to emphasize the significance of employing AI for predicting molecular mechanisms and structures, with the aim of enhancing the prospects of discovering novel drugs. Furthermore, they have explored the application of a comprehensive target activity approach to repurpose drug molecules by expanding the target profiles of drugs, encompassing potential therapeutic off-targets, thus facilitating the identification of new medical indications. Prasad and Kumar^27^ conducted a comprehensive survey of AI and ML techniques applied in diverse aspects of SARS-CoV-2 biochemistry, spanning from structural analysis to drug development. Their research provides a comprehensive examination of the present status of AI’s utilization in drug repurposing and pharmaceutical development. Pan et al.^28^ introduced guidelines for the effective utilization of deep learning methodologies and tools in the context of drug repurposing. First, they provided an overview of frequently employed bioinformatics and pharmacogenomics databases for the purpose of repurposing drugs. Subsequently, they delved into recently developed methods for representing data using both sequence-based and graph-based approaches, in addition to discussing the latest DL-based techniques. Additionally, their study explores the deployment of drug repurposing in combating the COVID-19 pandemic. Due to the persistently excessive costs associated with developing new drugs, the practice of repurposing already approved and investigational drugs is increasingly appealing. This is primarily due to the known safety profiles of these drugs and the potential reduction in cost barriers. It is worth noting that repurposing existing drugs for oncology has seen limited success, with ML and DL methods Playing an important part in making breakthroughs. In a review by F. Doshi-Velez^29^, various methods are explored, particularly in the context of cancer biology and immunomodulation, to uncover opportunities for repurposing drugs in the treatment of oncologic diseases.

From the literature, it can be concluded that few works have been developed in using ML and DL methods in drug repurposing. Most of the work done involves reviewing drug development processes as well as AI methods that could be used in drug repurposing, very few of the work presented has developed a drug repurposing AI model. Even though the number of XAI investigations is rapidly expanding, this paper considered the first to address the use of XAI with Vidarabine Features. However, there are several articles on XAI, but most of these articles concentrate on general XAI techniques and evaluation approaches. For example, in Naiem T. et al.^30^, the authors well-defined and evaluated interpretability. Their primary contribution is a taxonomy for evaluating interpretability. Abdul et al.^31^ constructed a citation network from a large corpus of explainability research, consisting of 289 core articles and 12,412 citing publications. However, their review focused on Human-Computer Interaction (HCI) research with an emphasis on explainability. Adadi et al.^32^ explored the motivations and outcomes of XAI research to highlight the significance of XAI.

This paper represents a novel and significant advancement in the field of drug repurposing with AI, specifically concerning vidarabine. While there have been limited literature reviews on the intersection of drug repurpose and AI, this paper stands out by integrating AI techniques to enhance the field and discover new features for compounds targeting anticancer cells. The main contributions of this paper are summarized as follows:

- Combination of DL with FRS theory, XAI, and medicinal chemistry to identify potential new drug candidates by analyzing the experimental results of Vidarabine natural product.
- The proposed model emphasizes the importance of uncovering hidden chemical features which are not discernible without the assistance of AI.
- The model’s predictions are validated through further anticancer assays against non-small lung cancer (A-549), human melanoma (A-375), and human epidermoid skin carcinoma (skin/epidermis) (A-431).
- Among the top ranked drugs based on the proposed model, sulfur and magnesium oxide was prioritized as both cheap and widely available compounds.

The subsequent sections of the paper follow this structure: Sect. 2 presents fundamental concepts, Sect. 3 provides an outline of the proposed model, Sect. 4 presents the discussion of obtained results, and Sect. 5 concludes the paper.

## Preliminaries

### Drug simplified molecular input line entry system (SMILE) format

SMILES is a method for representing molecule structure^33^ by employing ASCII strings. SMILES notation is valuable for computer-based analysis and the study of chemical properties. It adheres to specific rules for representing various structural properties of molecules. In SMILES notation, atoms are represented using combinations of alphabetic characters enclosed within square brackets, such as [Al] for Aluminum. In SMILES notation, bonds between atoms are indicated by special characters. Specifically, a single bond is typically represented by the character ‘-‘, while double bonds are denoted by ‘=’, triple bonds by ‘#’, and quadruple bonds by ‘$’. This notation helps describe the connectivity between atoms in a molecular structure^34^.

**Fig. 1:**
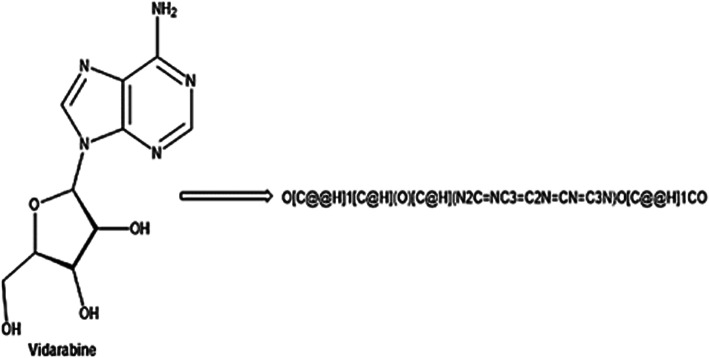
SMILE representation.

In SMILES notation, ring structures are represented by each ring being broken at any random moment to create an acyclic structure. Labels with a numerical ring closing are then added to indicate the interconnection of atoms that are not contiguous. For instance, let us consider dioxane, which is represented as O1CCOCC1 in SMILES notation. In this example, ‘1’ represents the ring present in the molecule. In cases where a single atom participates in multiple ring-closing bonds, multiple digits are used to indicate these bonds. such as the employment of an alternative SMILES notation for decalin could be C1CCCC2CCCCC12, whereby both ring-closing bonds involve the last carbon atom labeled as ‘1’ and ‘2’. This notation allows for the accurate representation of complex ring structures in molecules. Figure 1 represents an example of SMILES representation of Vidarabine.

### Fuzzy rough sets

The concept of fuzzy rough set (FRS) theory combines aspects of rough set theory and fuzzy set theory, drawing inspiration from their practicality and compatibility^35^. Within the framework of FRS theory, the connection between any two components inside the set is depicted using a fuzzy relation, as opposed to some correspondence. FRS theory introduced two operators known as fuzzy rough estimates for the upper and lower bounds, which are used to estimate a set based on fuzzy relations. To put it differently, FRS theory permitted the partial membership of an element in both upper and lower estimates, and it enabled the simulation of approximate equalities among elements through fuzzy relations.

Feature selection has garnered significant attention with the objective of choosing highly relevant features starting with unprocessed datasets to enhance how well a learning model performs. FRS entails the process of selecting a subset of features from the original raw dataset for subsequent learning tasks, as highlighted in previous studies^36,37^.

FRS theory serves as a potent mathematical approach for feature selection. Building upon this foundation, feature selection methods employing FRS have been thoroughly examined and studied from a theoretical perspective in various works^38^.

Given a fuzzy relation *R* on the universal set *U* and a fuzzy set *A* defined on *U*, the upper and lower approximations of *A* can be represented as a pair of fuzzy sets on *U*, as follows:

1 $$a \underline{p}{r}{R} (A)= {\text{sup}} \{A(y) | y \in [x]{R} \},x \in U,$$

2 $$\underline{apr_{R}(A)} = inf \{A(y) | y \in [x]_{R}\}, x \in U,$$

where $\:{\left[x\right]}_{R}$ is the equivalence class of element *x* based on *R*. If Eq. (1) is not equal to Eq. (2), then *A* is a FRS of *U*, where $\underline{apr_{R}(A)}$ is the fuzzy rough lower approximation of *A*; $a \underline{p}{r}{R} (A)$is the fuzzy rough upper approximation of *A*. Additionally, the properties of operations on FRSs are the same as those on rough sets.

### Convolutional neural network (CNN)

**Fig. 2:**
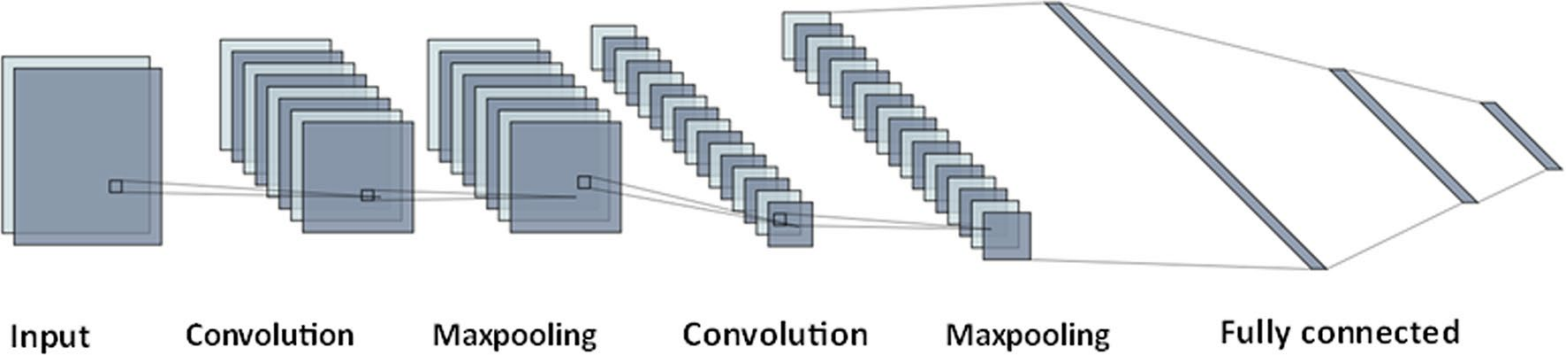
The fundamental architecture of CNN.

The Standard CNN^2,39^ which is an evolution of the Multilayer Perceptron (MLP) neural network represents a significant advancement tailored for the processing of data in a two-dimensional format. Like its neural network counterparts, the CNN comprises neurons with activation functions, biases, and weights. CNN can autonomously acquire layered data representations, which are more durable and descriptive compared to manually crafted features. It consists of numerous layers of neurons. Within the convolutional layer, a group of kernels or filters is employed on the input data to produce feature maps which encompass diverse characteristics of the input. The pooling layers decrease the size of the feature maps by keeping the most vital traits, whereas the activation functions add nonlinear qualities to the result of every convolutional layer. At the end, the fully connected layers use the results from the earlier stages to conduct the last regression or categorization. Figure 2 gives a crucial visual explanation of the main components of a CNN. This clarifies how the CNN structure takes out features from input data and conducts regression using many pooling and convolutional layers, along with fully connected layers.

## The proposed framework

**Fig. 3:**
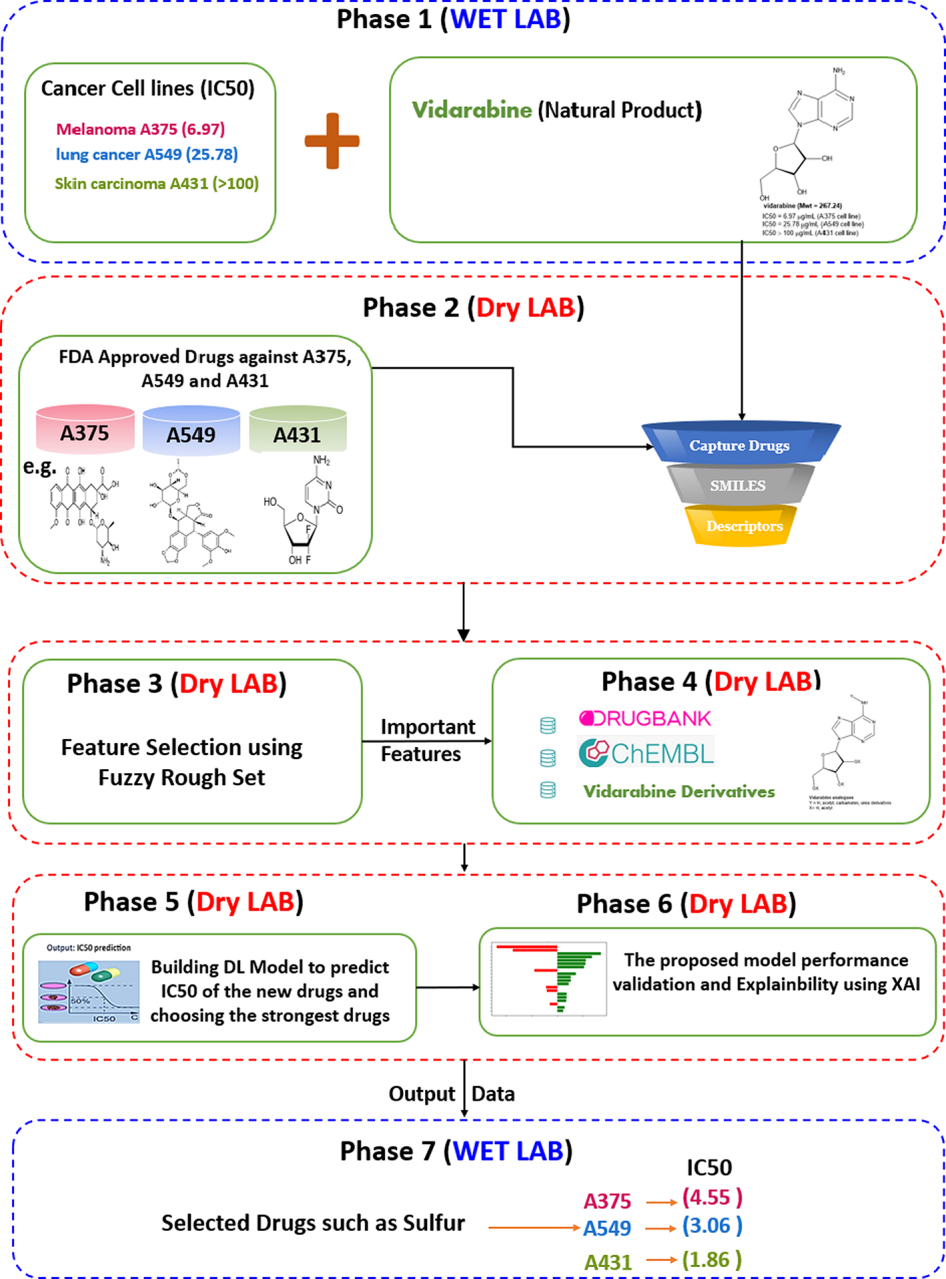
The Framework of the Proposed Model.

There are seven phases in the proposed framework, as seen in Fig. 3. Each phase will be explained in detail in the following subsections.

### Phase 1: Anticancer studies of Vidarabine

This phase presents the materials and methods used to make the anticancer studies of Vidarabine (WET LAB). The effect of Vidarabine against different cancer cell lines is calculated by the determination of IC_50_. These cell lines are A-549 (Non-small Lung cancer), A-375 (Human Melanoma), and A-431(Human epidermoid skin carcinoma skin/epidermis). Furthermore, its CC_50_ against normal cells was calculated. These cell lines are OEC (Oral epithelial cell) and HSF (Human skin fibroblast). Having the IC_50_ and CC_50_, the selective index (SI) was calculated to determine the compound’s safety.

#### Anti-cancer studies (in vitro assay)

Vidarabine was purchased from Masaaki Co., Ltd. Fukuoka West office. Address: 723 − 13 Tomari, Itoshima, 819–1111 Japan. Regarding Cell Culture, cell lines namely A-549, A-375, A-431, OEC, and HSF cells were obtained from Nawah Scientific Inc. located in Mokatam, Cairo, Egypt.

The cells were grown in RPMI medium supplemented with 100 mg/mL streptomycin, 100 units/mL penicillin, and 10% heat-inactivated fetal bovine serum. They were incubated at 37 °C in a humidified environment containing 5% CO_2_^40^.

For the cytotoxicity assay, cell viability was measured using the sulforhodamine B (SRB) assay. A 100 µL suspension containing 5 × 10^3^ cells was seeded into 96-well plates and incubated in a complete medium for 24 h. The cells were then treated with 100 µL of medium containing the test samples at varying concentrations (0.01, 0.1, 1, 10, and 100 µg/mL). After 72 h of exposure, the medium was replaced with 150 µL of 10% trichloroacetic acid to fix the cells, which were incubated at 4 °C for 1 h. The trichloroacetic acid solution was then discarded, and the cells were rinsed five times with distilled water. Following this, 70 µL of SRB solution (0.4% w/v) was added, and the cells were incubated in the dark at room temperature for 10 min. The plates were then washed three times with 1% acetic acid and left to air-dry overnight. To dissolve the protein-bound SRB stain, 150 µL of TRIS (10 mM) was added, and absorbance was measured at λ_max_ 540 nm using a BMG LABTECH-FLUOstar Omega microplate reader (Ortenberg, Germany). The experiment was carried out in 3 replicates^41^.

#### Cell cycle arrest

This assay was carried out for Sulfur on A-375 cells and Magnesiun on A-431 cells. After treating the cells (10^5 cells) with the test compounds for the specified time, they are harvested through trypsinization and washed twice with ice-cold PBS (pH 7.4). The cells are then resuspended in 2 mL of 60% ice-cold ethanol and incubated at 4ºC for 1 h for fixation. After fixation, the cells are washed twice as much with PBS (pH 7.4) and resuspended in 1 mL of PBS containing 50 µg/mL RNAase A and 10 µg/mL propidium iodide (PI). Following a 20-minute incubation in the dark at 37ºC, the cells are analyzed for DNA content using flow cytometry (ACEA Novocyte flow cytometer, ACEA Biosciences Inc., San Diego, CA, USA) with the FL2 signal detector (λex/em 535/617 nm). For each sample, 12,000 events are recorded, and cell cycle distribution is calculated using ACEA NovoExpress software (ACEA Biosciences Inc., San Diego, CA, USA)^42^.

#### Apoptosis/necrosis assessment using flow cytometry

This assay was carried out for Sulfur on A-375 cells and Magnesiun on A-431 cells to confirm their cytotoxic activity suggested. Apoptotic and necrotic cell populations are assessed using the Annexin V-FITC apoptosis detection kit (Abcam Inc., Cambridge Science Park, Cambridge, UK) in conjunction with two-channel flow cytometry. Following a 48-hour treatment with sulfur and magnesium, the cells (10^5 cells) are harvested via trypsinization and washed twice with ice-cold PBS (pH 7.4). The cells are then incubated in the dark for 30 min at room temperature with 0.5 mL of Annexin V-FITC/PI solution, following the manufacturer’s protocol. After staining, the cells are processed through an ACEA Novocyte flow cytometer (ACEA Biosciences Inc., San Diego, CA, USA) and analyzed for FITC and PI fluorescent signals using the FL1 and FL2 signal detectors, respectively (λex/em 488/530 nm for FITC and λex/em 535/617 nm for PI). A total of 12,000 events are recorded per sample, and FITC and/or PI-positive cells are quantified through quadrant analysis using ACEA NovoExpress software (ACEA Biosciences Inc., San Diego, CA, USA)^42^.

#### Statistical analysis

Data is presented as the mean ± standard deviation (SD) for all measured parameters. Data analysis was conducted using the Graph Pad Prism Data Analysis software, employing multiple comparisons. Additionally, a one-way analysis of variance (ANOVA) test was employed.

### Phase 2: Data collection and preprocessing

**Fig. 4:**
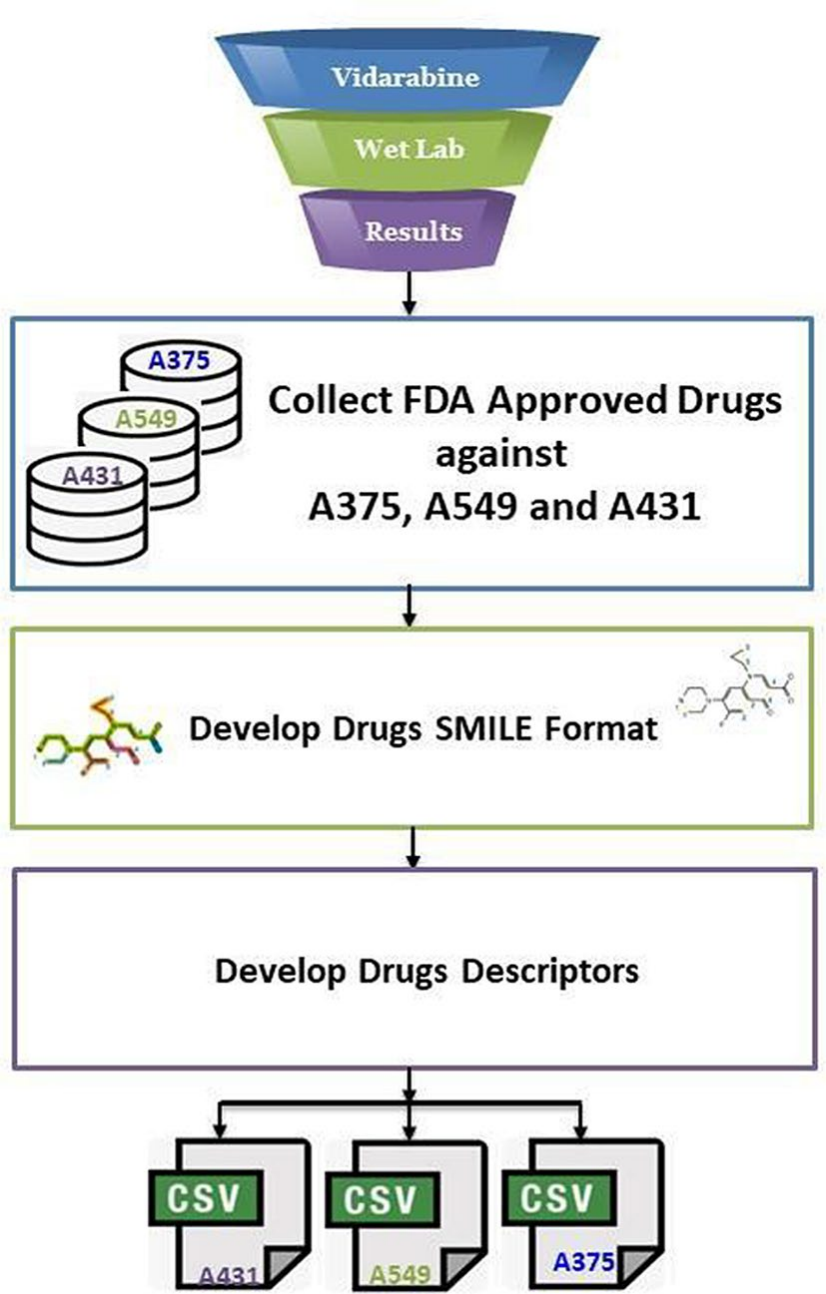
The process of data collection and preprocessing.

This phase presents the process of how the dataset is collected and preprocessed to address the problem examined in this research. Figure 4 presents the process of data collection and preprocessing. Vidarabine was used as a lead compound in the data sets for building the AI model^10,43^. The dataset was constructed from standard drugs of each cell line (A-375, A-549, A-431) with their IC_50_ and it is collected from cancerrxgene database^41^. The Vidarabine WET LAB results obtained from phase 1 considered three cell lines: A-375, A-549, and A-431 were incorporated in each dataset correspondingly. Then, the SMILE formats of the collected drugs beside the Vidarabine are developed for each cell line using the PubChem database^44^ and DrugBank database^45^. Afterwards, the descriptors or features for each drug are developed using RDKIT which is an open-source toolkit for cheminformatics and ML^46^. Finally, the data set is gathered and saved in CSV files. A sample of one of these files is presented in Table 1. These CSV files play as the input data for phase 3.

### Phase 3: feature selection using fuzzy rough set

The main objective of phase 3 is to choose the most important descriptors or features of the investigated drugs obtained from the CSV output files in phase 2 while achieving the best IC_50_ values. In the prediction of IC_50_ values, the task of feature selection involves recognizing descriptors in an automated way that hold the highest relevance regarding a drug’s inhibitory concentration against a particular target molecule. The selected descriptors should capture essential molecular characteristics contributing to the observed IC_50_ values. These descriptors could pertain to structural attributes, chemical properties, Morgan fingerprint that represent molecular structure in binary format for similarity analysis, or any other physicochemical properties that play a pivotal role in the drug’s efficacy. Therefore, the FRS has been employed for feature selection in this phase.

The model uses fuzzy membership functions to compute the Fuzzy lower and upper approximations to determine the impact of feature subsets on the prediction of the target variable, as well as to evaluate dependencies to identify significant features. Equations 3, 4, and 5 lead the procedure for fuzzy lower approximation $\:{S}_{L}^{Y}$ and fuzzy upper approximation $\:{S}_{U}^{Y}$, taking membership values into account. Reduced selection ($\:R$) selects significant feature subsets as indicated in the following Eq. 

3 $$\:{S}_{L}^{Y}=\left\{v\:|\:\mu\:\:S\left(v\right)\:\ge\:\:\mu\:\:Y\left(v\right)\:\right\}$$

4 $$\:{S}_{U}^{Y}=\left\{V\:|\:\mu\:\:S\left(v\right)\:>\:\mu\:\:Y\left(v\right)\:\right\}$$

Where $\:\mu\:\:S\left(v\right)\:$signifies the degree of membership in the target variable v in the set $\:S$ and $\:\mu\:\:S\left(v\right)$ represents the degree of membership in the target v for the feature subset $\:Y$. The reduced selection is given by:

5 $$\:R=\left\{S\:\subseteq\:U\:|\:{S}_{L}^{Y}\ne\:{S}_{L}^{{Y}^{{\prime\:}}}\forall\:\:{Y}^{{\prime\:}}\:\subset\:Y\:\right\}$$

Where $\:Y$ is some input features, $\:U$ represents the universe of input features.

**Table 1:**
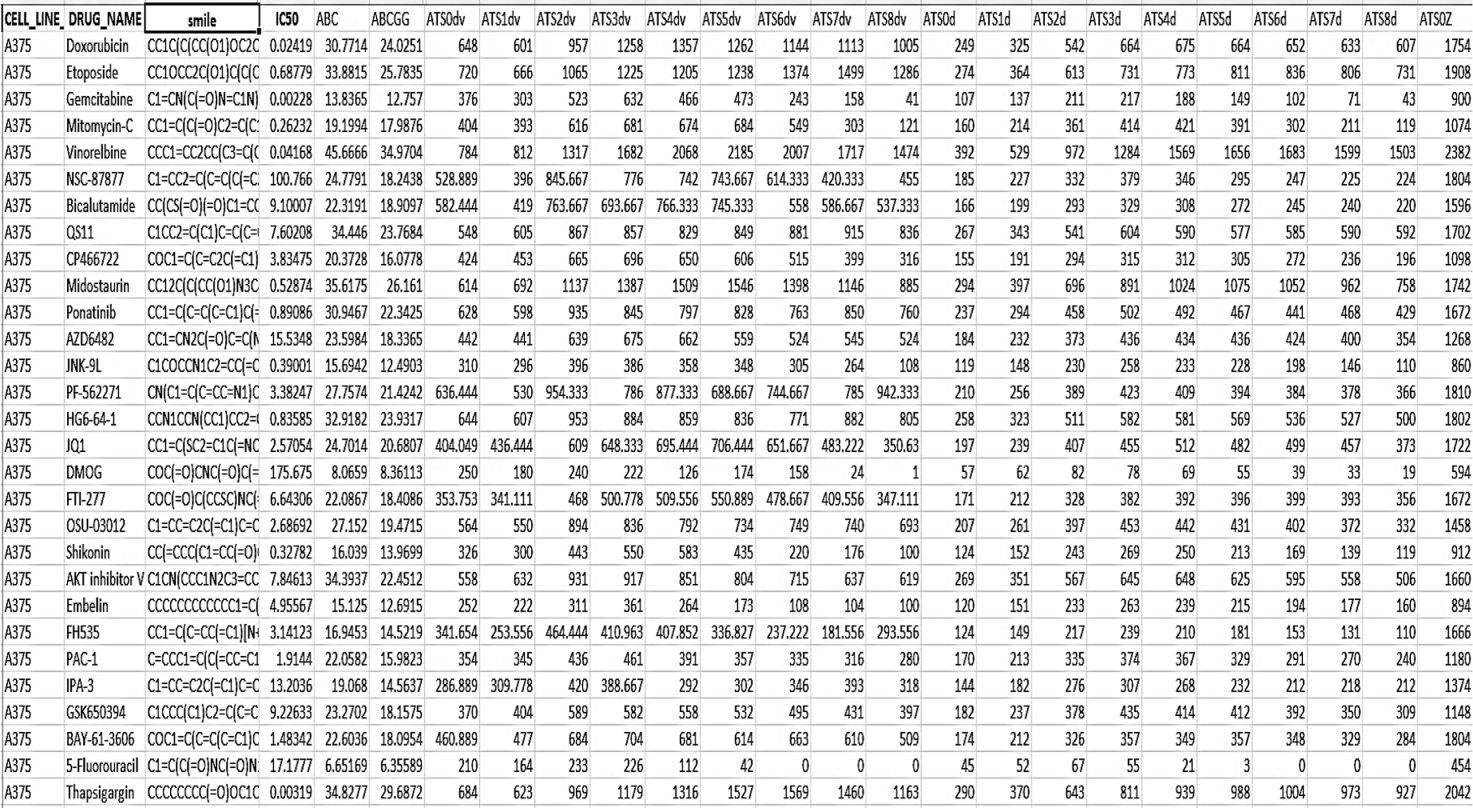
A sample of the collected dataset of the cell line A-375.

Table 2 provides a summary of the generated and selected descriptors for cell lines, while Table 3 indicates the best descriptors (features) selected by the FRS algorithm that contribute to the prediction of IC_50_ values against the three-cancer cell lines; A-549, A-375, and A-431. The table contains the most important descriptors which affect the IC_50_ prediction values for two cases: using and not using the Vidarabine in the prediction. That is to help in the drug repurpose process in the next phase of the proposed model.

**Table 2:** Summary of Generated and selected descriptors for each cancer cell line.

Cell Lines	Number of the generated descriptors	Number of the selected descriptors using FRS algorithm
A-549	441	9
A-375	442	9
A-431	442	9

**Table 3:** Selected descriptors using FRS algorithm for each cancer cell line.

Cell Lines	The selected descriptors without using Vidarabine	The selected descriptors using Vidarabine
A-549	ATSC4dv, ATSC1m, ATSC7are, Xch-6d, SssO, fMF, PEOE_VSA3, SMR_VSA7, EState_VSA4	MATS1i, Mp, SaaN, JGI10
A-375	AATS0dv, ATSC3v, GATS2dv, Xch-6d, SdCH2, SssNH, SdS, IC2, EState_VSA2	AATS1dv, MATS1i, SMR_VSA7, piPC8, JGT10
A-431	SsNH2, ZMIC3, PEOE_VSA4, PEOE_VSA5, PEOE_VSA11, SMR_VSA4, EState_VSA2, EState_VSA9, SRW07	AATS0dv, AATSC2pe, AATSC1are, GATS1i

### Phase 4: Drug repurposing based on previous results (from phase 3)

To predict the IC_50_ and pick up the best drug candidate, the new input data sets were constructed from (a) suggested Vidarabine derivatives (supplementary file 1), (b) nucleotides drugs (obtained from DrugBank), (c) and other reported compounds from Compile. The criteria for selecting these compounds are according to the outcomes of the prior phase in Table 3. Most of the above common features are related to specific physicochemical properties such as molecular weight, molecular polarizability, and topological structure.

### Phase 5: Deep learning prediction model

**Fig. 5:**
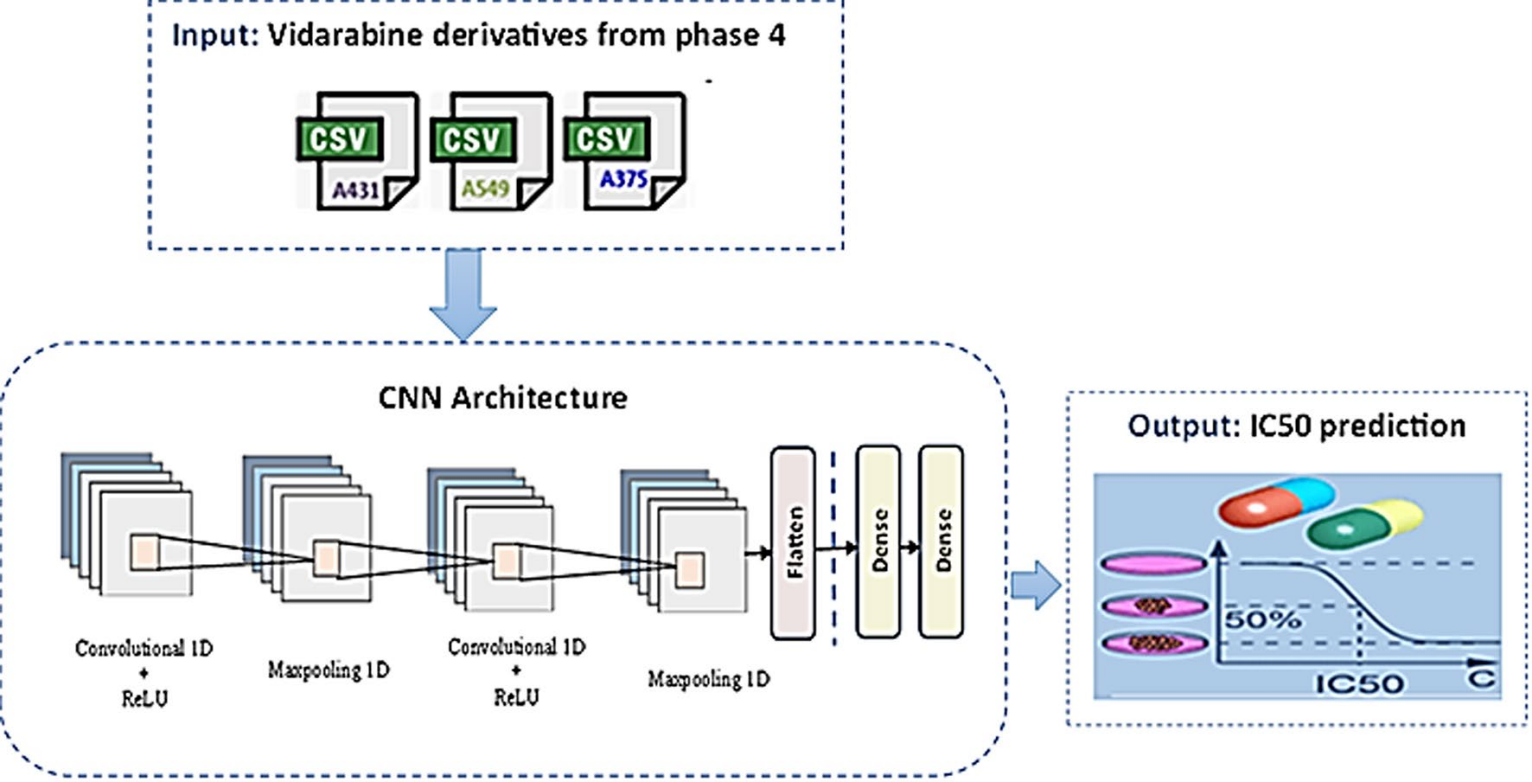
The proposed CNN architecture.

In the intricate realm of drug discovery, the pivotal mission of predicting IC_50_ values emerges as a critical endeavor. This endeavor involves evaluating a drug’s inhibitory concentration against a specific target molecule, a task with profound implications for pharmaceutical development. To tackle this challenge, CNN architecture has been meticulously crafted. The CNN model, shown in Fig. 5, was trained using descriptors obtained from phase 4 for the new compounds based on Vidarabine features against each cancer cell line. Each compound is represented by SMILE notation which is then converted into descriptors for model training. The CNN model comprises of a one-dimensional convolutional input layer with a kernel size of 3, and a ReLU activation function followed by a MaxPooling1D layer that is a pooling layer with a default pool size of 2; and the second Conv1D layer with a kernel size of 3, and ReLU activation, and then another MaxPooling1D layer. The output from the convolutions is flattened into a 1D vector, which is then passed through two Dense layers: one with 64 neurons and ReLU activation, and an output layer with 1 neuron for regression. The proposed architecture serves the paramount objective of predicting IC_50_ values, facilitating the evaluation of new drugs, and the identification of potentially potent candidates for further exploration.

### Phase 6: Evaluating the performance of the DL model and explainability using XAI

#### Evaluating the DL model’s performance

After the training phase of the proposed model, it is imperative to conduct verification and testing procedures. The effectiveness of the proposed DL prediction model is validated through the application of diverse performance assessment metrics.

Mean Square Error (MSE)^47^ calculates the squared disparity between predicted values and actual parameters, expressed as the mean in Eq. 6. A lower MSE indicates superior forecasting model performance.

6 $$\:MSE=\frac{\sum\:{({y}_{k}-\widehat{{y}_{k}})}^{2}}{m}$$

Where $\:{y}_{k}$ is the ith observed value, $\:\widehat{{y}_{k}}\:$is the corresponding forecasted value, and $\:m$ is the number of observations.

Mean Absolute Error (MAE) quantifies the average absolute difference between real and forecasted values generated by a regression model^47^, as indicated in Eq. 7.

7 $$\:MAE=\:\frac{\sum\:|{y}_{k}-{x}_{k}|}{m}$$

where: $\:m$ is the total number of observations, $\:{y}_{k}$ is the real value for the k^th^ observation, $\:{x}_{k}$ is the forecasted value for the k^th^ observation.

The performance of the proposed DL model was evaluated. It is utilized to forecast the IC_50_ values of Vidarabine against the three distinct cancer cell lines: A-549, A-375, and A-431. This is followed by a detailed comparison between the outcomes generated by the DL model with the results derived from Pharmacological Studies presented in Phase 1. This comparative analysis was conducted to gain insights into the model’s performance and its alignment with established pharmacological findings.

#### XAI insights: interpretable explanations for drug potency prediction

Recent research has introduced novel methods for enhancing the explainability of DL models^48^, enabling more interpretable and accurate explanations for their predictions. Explainable artificial intelligence (XAI) is a set of approaches and processes that allow consumers to understand and trust the outcomes produced by machine learning algorithms. XAI is used to explain an AI model’s expected impacts and potential biases. It helps to analyse the model’s accuracy and promotes transparency. XAI assists organisations in adopting a responsible approach to AI development by either (i) creating white- or Gray-box ML models that are interpretable by design (at least to some extent) while achieving high accuracy, or (ii) providing black-box models with a minimum level of interpretability in the event that white- or Gray-box models are unable to achieve an admissible level of accuracy, eXplainable Artificial Intelligence (XAI) technology Working with DNN models and figuring out how to make their outputs comprehensible to people, XAI approaches are vital^3,49^.

In addition, we can try to elucidate the ML model using the phrases (i) interpretability and (ii) explainability. Interpretability increases developers’ trust in their ability to understand where the model draws its findings by allowing them to dive into the model’s decision-making process.

In the context of this paper, the capabilities of the proposed DL model are extended by incorporating the Local Interpretable Model-agnostic Explanations (LIME) model^50^. LIME effectively elucidates the inferences made by DL models through a localized approximation of the inference point. This algorithm constructs a linear regression model in the vicinity of a particular inference point which requires clarification. Positive-weighted features in the linear regression model support the prediction decisions, whereas features with negative weights challenge those decisions. In addition, LIME is used to further elucidate the findings, providing interpretable explanations for the selection of the top-ranked compounds.

### Phase 7: Anticancer studies of the selected agents (WET LAB)

In this phase, the anticancer studies of the selected agents by the proposed AI model are conducted to validate the DRY LAB results of AI. All results are presented in the next section.

## Results and discussion

### Results of the Anticancer studies (phase 1 in the proposed model)

#### In-vitro cytotoxicity assay

**Fig. 6:**
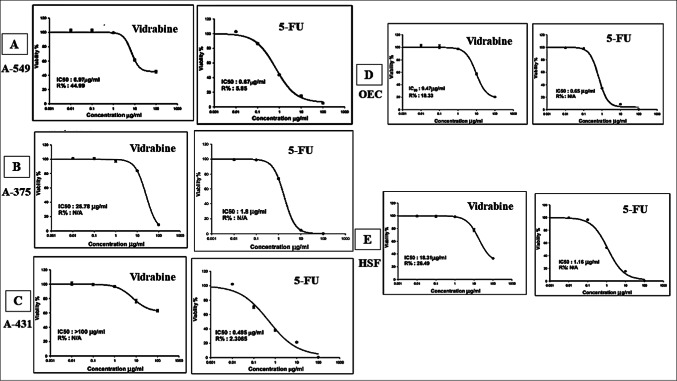
In-vitro SRB cytotoxicity assay (IC_50_) of Vidarabine and 5-FU against **(A)**: A-549 and **(B)**: A-375, **(C)**: A-431, **(D)**: OEC, **(E)**: HSF cell lines in increasing concentrations (0.01, 0.1, 1, 10 and 100 µg/mL). Data points are expressed as mean ± SD (*n* = 3).

The in vitro cytotoxic activity IC_50_ for Vidarabine alkaloid was assessed and results are listed in Table S1(supplementary table) and Fig. 6. The cytotoxicity was evaluated against three cancer cell lines, namely A-549, A-375, and A-431, and two normal cell lines namely OEC, and HSF. The assay was conducted using SRB assay for IC_50_ calculation 5 different concentrations 0.01, 0.1, 1, 10, and 100 µg/mL compared to 5-Fluorouracil (5-FU) as a positive control. The results of the cytotoxicity activity revealed that it exhibited selective potent cytotoxicity on A-549 cancer cells followed by A-375 and then A-431. Besides, the IC_50_ on OEC and HSF were 9.47 and 18.31 µg/mL, respectively.

**Fig. 7:**
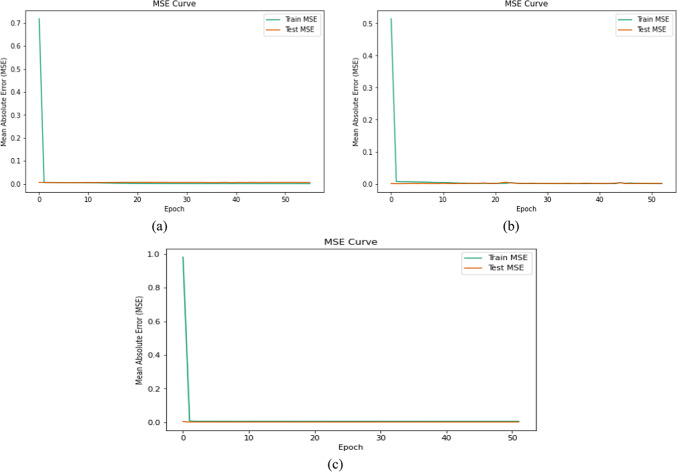
MSE comparison for testing and training datasets to predict IC50 on the three different cell lines. (**a**) model MSE for the A-549 cell line. (**b**) model MSE for the A-375 cell line. (**c**) model MSE for the A-431 cell line.

**Fig. 8:**
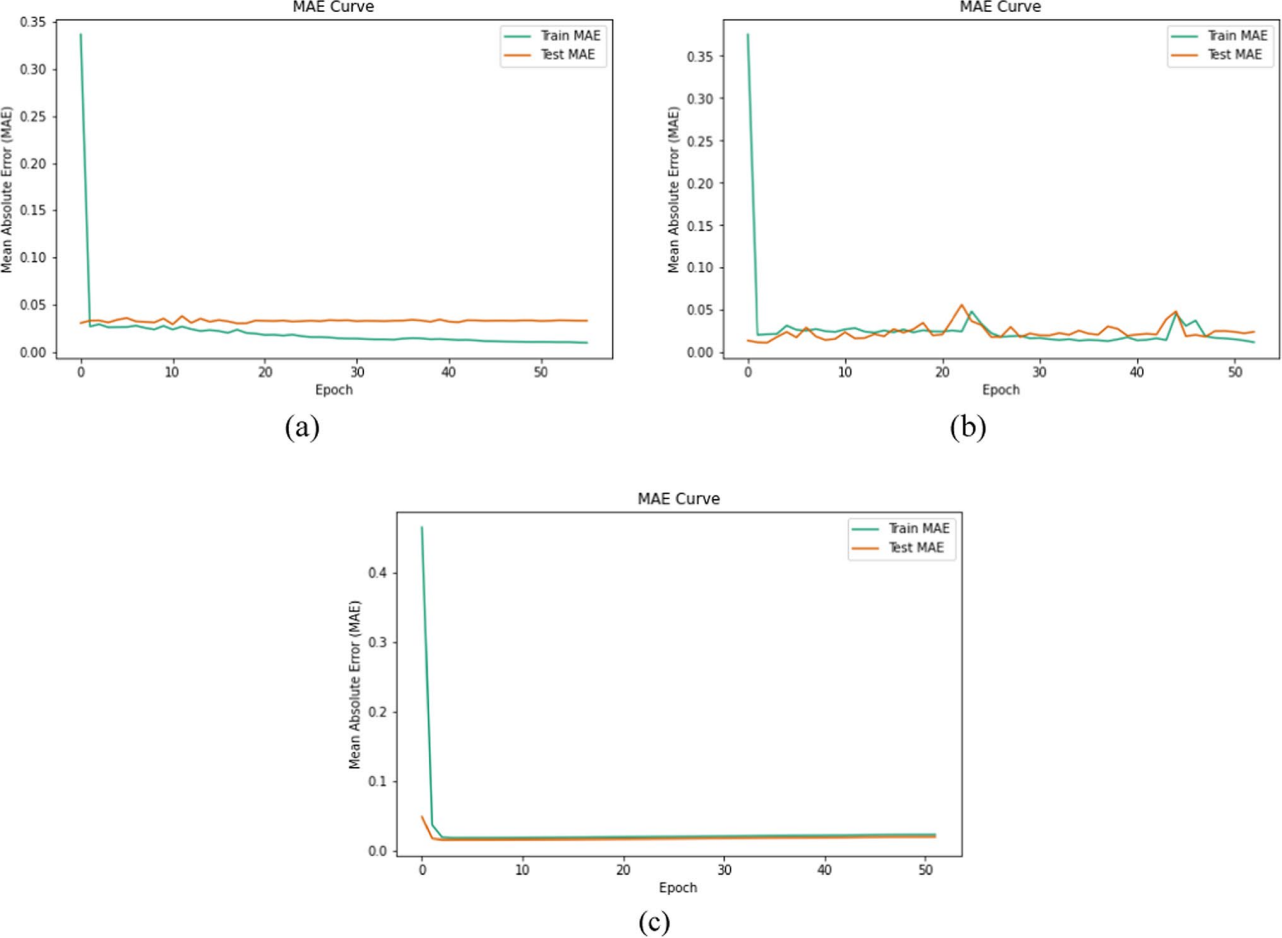
MAE comparison for testing and training datasets to predict IC50 on three different cell lines. (**a**) model MAE for the A-549 cell line. (**b**) model MAE for the A-375 cell line. (**c**) model MAE for the A-431 cell line.

The optical microscope-stained images were recorded as shown in Figs. 7S and 8S (at the supplementary file) comparing the effect of Vidarabine and 5-FU at the same concentration levels. Figures show several morphological differences in comparison to the (-ve control).

### IC_50_ prediction for FDA-approved drugs using the proposed CNN regression model (phase 5 in the proposed model)

The CNN regression model is employed for IC_50_ prediction against the three investigated cancer cell lines, which are A-549, A-375, and A-431. Table 4 shows the performance comparison for predicting IC_50_ on different cell lines in terms of MSE and MAE. Although the CNN regression model demonstrates better performance across all three cell lines, it gives the highest performance for the A-375 cell line shown by extremely low MSE (0.000194) and MAE (0.011517).

**Table 4:** CNN performance comparison for predicting IC_50_ on the three different cell lines.

Cell Line	MSE	MAE
A-549	0.004813	0.035583
A-375	0.000194	0.011517
A-431	0.000995	0.016873

The MSE and MAE comparison for the testing and training datasets to predict IC_50_ on the three different cell lines are given in Figs. 7 and 8, respectively.

### Exploring Vidarabine IC_50_: a comparative analysis of DL model and pharmacological studies (phase 6 in the proposed model)

This section compares the results of the proposed DL model (DRY LAB) to the findings of the WET LAB to validate its efficacy. The suggested model predicts the IC_50_of Vidarabine against three cancer cell lines. Table 5 presents the comparison of the results of the DL model and the results of Pharmacological Studies shown in Sect. 4.1. It is observed from Table 4 that the correlation between the measured IC_50_ and predicted IC_50,_ especially for A-549 and A-375 cell lines is better than A-431.

**Table 5:** Vidarabine IC_50_ comparison between the DL model prediction and pharmacological studies.

Cell Line	DRY LAB	WET LAB
A-549	5.09	**6.97**
A-375	24.27	**25.78**
A-431	60.58	**> 100**

### IC50 prediction with CNN regression: evaluating New drugs and identifying potent candidate

Once the performance of the proposed DL model has been evaluated and validated, it is employed to predict the IC_50_ values for novel drugs concerning the three distinct cancer cell lines. Subsequently, the most potent drugs have been selected which possess the lowest IC_50_ values among the pool of candidate drugs. Tables 6, 7 and 8 show the predicted IC_50_ of the most potent drugs for the three distinct cancer cell lines.

**Table 6:** The predicted IC50 of the most potent drugs for the A-549 cell line.

Drug_Name	IC_50_
Magnesium oxide	**0.006296**
Iofetamine	0.006341
Silver nitrate	0.006341
Liothyronine sodium	0.006341
Cisplatin	0.006341
Aluminum hydroxide	0.006341
Pentetate calcium trisodium yb 169	0.006341
Liothyronine	0.006341
Calcium carbonate	0.006341
Carboplatin	0.006341
Sodium stibogluconate	0.006341
Hydrotalcite	0.006341
Iobenguane	0.006341
Oxaliplatin	0.006341
Cisplatin	0.006341
Sodium iodide	0.006341
Halothane	0.017352
Magaldrate	0.128526
Magnesium carbonate	0.369054

**Table 7:** The predicted IC50 of the most potent drugs for the A-375 cell line.

Drug_Name	IC_50_
Sulfur, precipitated	**0.357777**
Magnesium oxide	**0.972352**
Aluminum hydroxide	1.475211
Magnesium carbonate	2.146655
Cisplatin	2.749105
Cisplatin	2.749105
Acetic acid	2.834914
Lithium carbonate	3.341439
Calcium carbonate	3.834431
Glycine	3.935773
Sodium iodide	3.956215
Silver nitrate	4.27554
Magaldrate	4.314857
Sodium lactate	6.345456
Phenol	6.406353
Halothane	7.084631
Carboplatin	7.517887
Niacinamide	8.045052
Isoflurane	9.004047

**Table 8:** The predicted IC50 of the most potent drugs for the A-431 cell line.

Drug_Name	IC_50_
Sulfur, precipitated	**2.398669**
Magnesium oxide	**2.999902**
Aluminum hydroxide	5.856307
Magnesium carbonate	7.400445
Lithium carbonate	10.15839
Acetic acid	10.72898
Calcium carbonate	12.9502
Glycine	14.02106
Magaldrate	14.03163
Sodium lactate	21.25496
Phenol	21.71362
Halothane	24.47713
Isoflurane	25.27882
Enflurane	25.68701
Niacinamide	25.7594
Silver nitrate	26.46598
Salicylic acid	28.70219
Tiopronin	31.55554
Aminosalicylic acid	31.85452

To move to the next step, the top ranked compounds illustrated in the previous three tables were selected. Among them, the simplest and cheapest ones were prioritized for the next WET LAB phase. Sulfur and magnesium oxide (MgO) are very well-known pharmaceutical compounds.

Sulfur element is frequently used in Asia as a herbal medicine to treat inflammation and cancer^51^.

### XAI insights: interpretable explanations for drug Potency Prediction (results of phase 6)

**Fig. 9:**
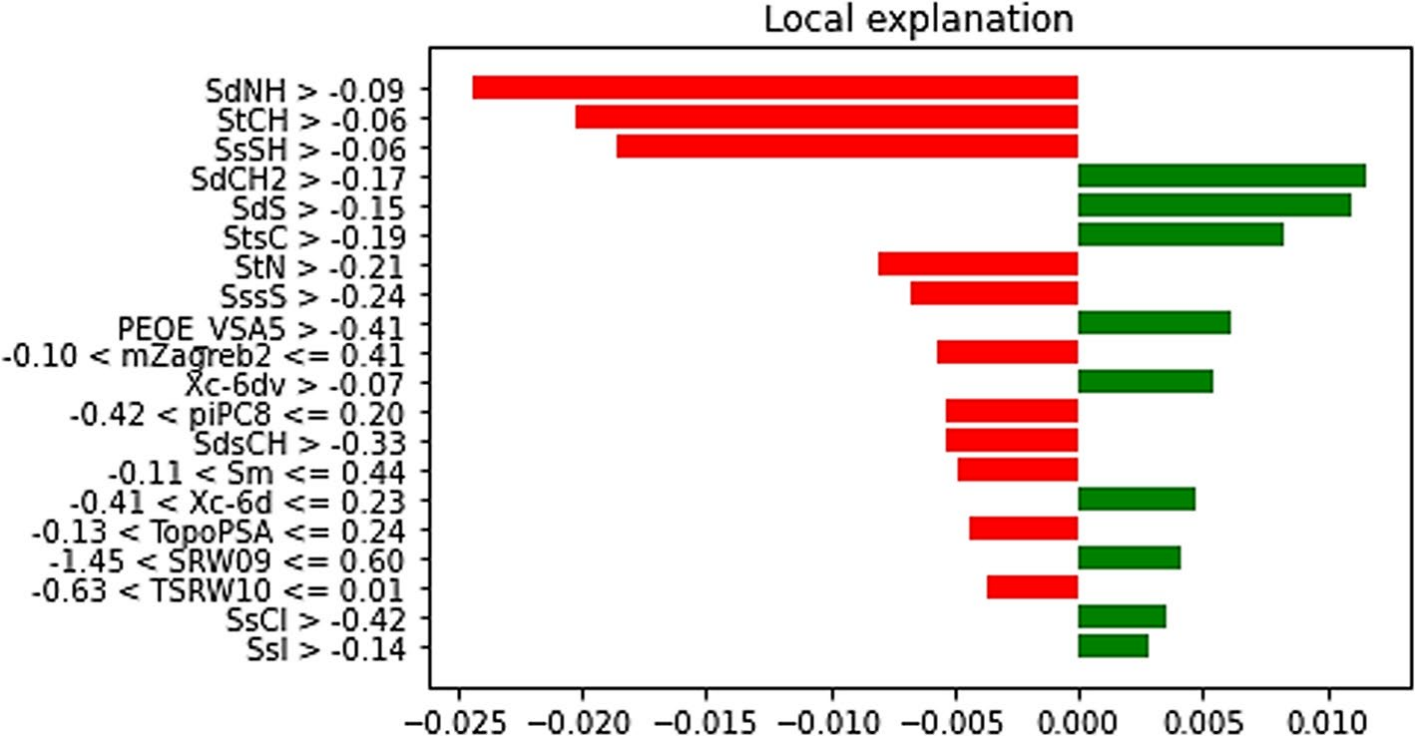
Interpretable Explanations for Compound Selection and Feature Significance in IC_50_ Predictions for A-549 Cell Line.

The LIME model prioritizes the simplest and most cost-effective compounds, such as magnesium oxide (MgO), which are well-established pharmaceutical agents. Figure 9 provides the significance of each feature and how they collectively contribute to the IC_50_ predictions for the A-549 cell line. Each feature is accompanied by a weight, representing its influence on the prediction where the green bars illustrate the features that make the prediction stronger, whereas the red bars show features that provide evidence against it. Features like SdCH2, SdS, StsC, and PEOE_VSA5 have notable positive contributions, indicating their importance in shaping IC_50_ predictions for A-549 cell lines. Conversely, features such as SdNH, StCH, SsSH, StN, SssS, -0.10 < mZagreb2 < = 0.41, -0.42 < piPC8 < = 0.20, SdsCH, -0.11 < Sm < = 0.44, -0.41 < Xc-6d < = 0.23, -0.13 < TopoPSA < = 0.24, -1.45 < SRW09 < = 0.60, -0.63 < TSRW10 < = 0.01, SsCl, and SsI exhibit negative effects on IC_50_ predictions.

**Fig. 10:**
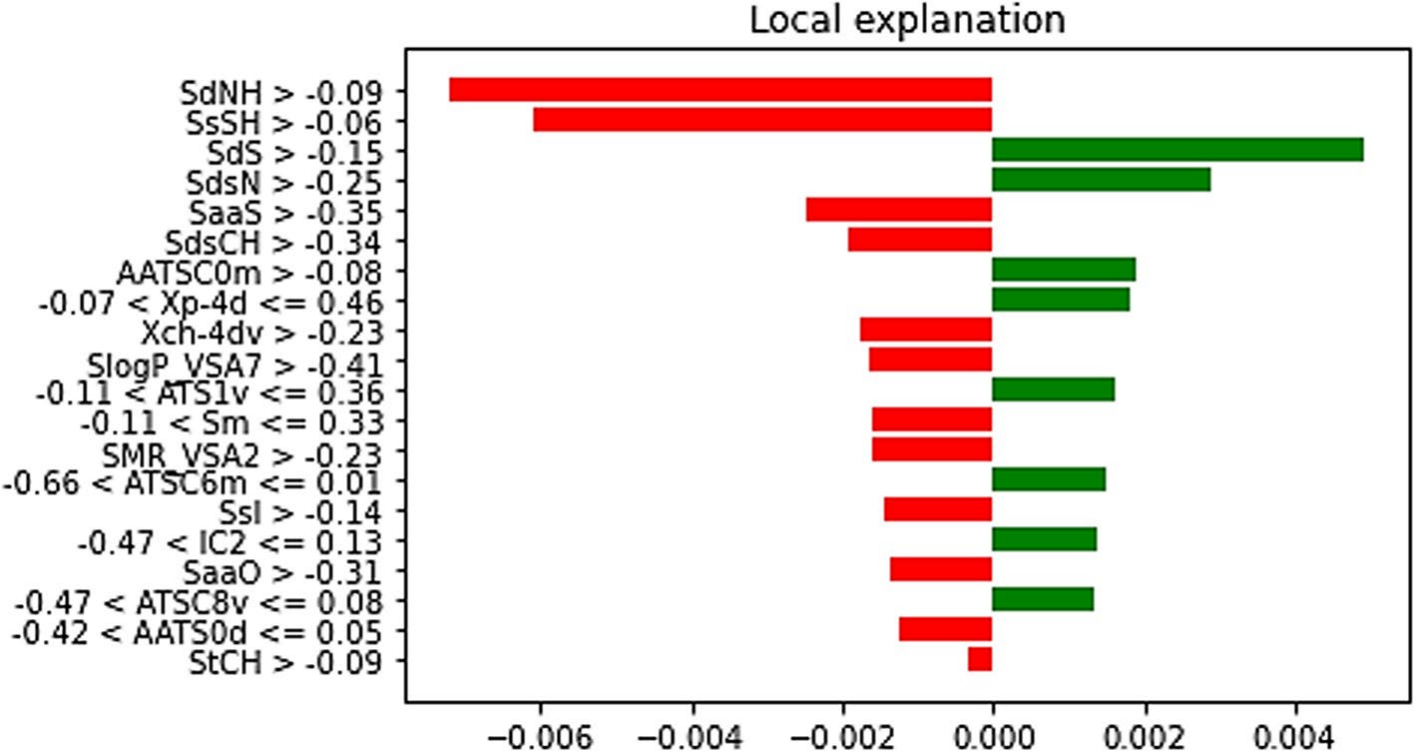
Interpretable Explanations for Compound Selection and Feature Significance in IC_50_ Predictions for A-375 Cell Line.

For the A375 cell line, as shown in Fig. 10, features such as SdS, AATSC0m, ATS1v, and ATSC6m displayed positive contributions to IC_50_ predictions, while features like SdNH, SsSH, SaaS, and SdsCH had adverse effects.

**Fig. 11:**
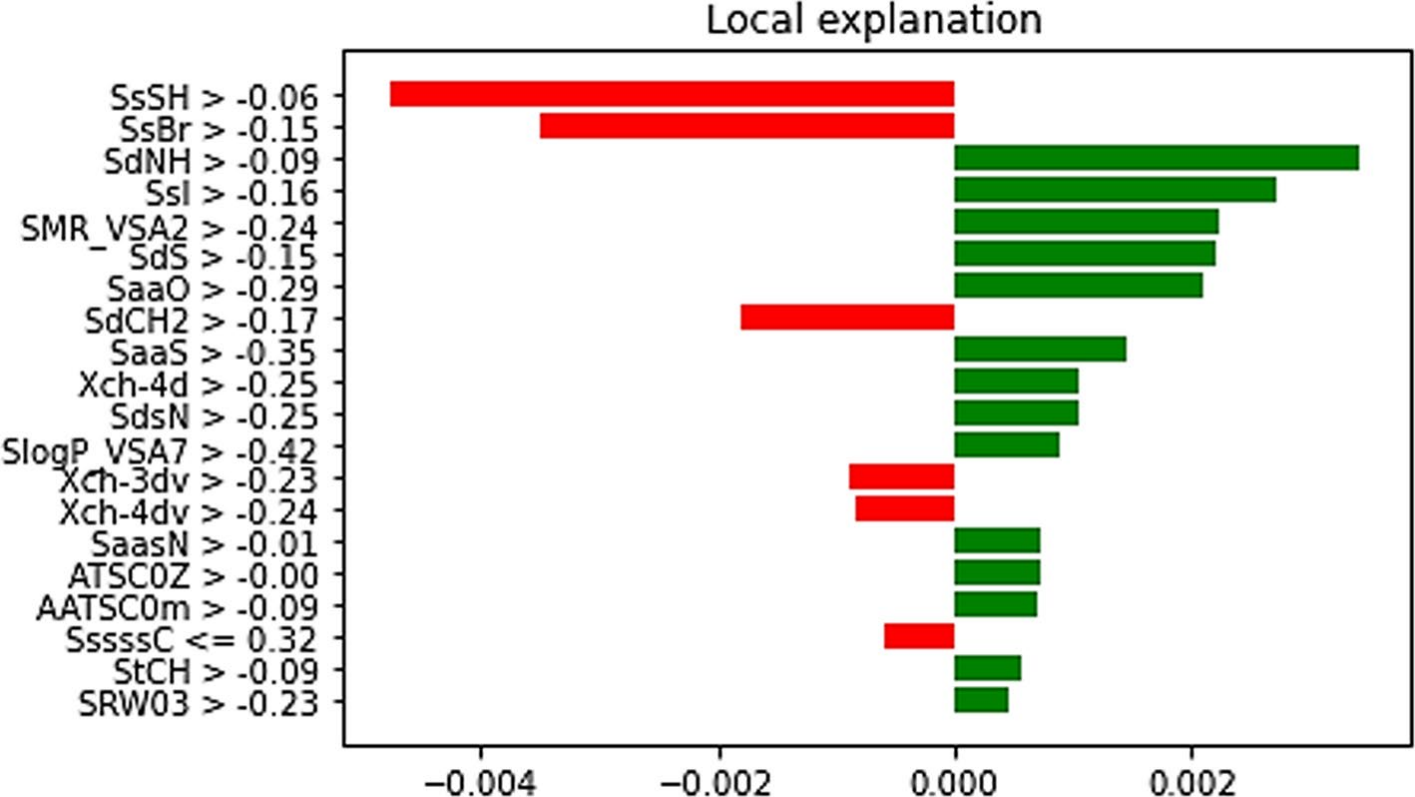
Interpretable Explanations for Compound Selection and Feature Significance in IC50 Predictions for A-431 Cell Line.

For the A-431 cell line, as shown in Fig. 11, features such as SdNH, SsI, and SMR_VSA2 exhibit positive contributions to the predictions, with positive weights ranging from 0.0022 to 0.0034. In contrast, features like SsSH, SsBr, and SdCH2 have negative weights, suggesting adverse effects on predictions. Other features, including SaaS, Xch-4d, and SdsN, make modest positive contributions with weights around 0.001.

### Anticancer studies for sulfur and magnesium oxide (WET LAB -phase 7 in the proposed model)

#### Cytotoxicity for sulfur and magnesium oxide

**Fig. 12:**
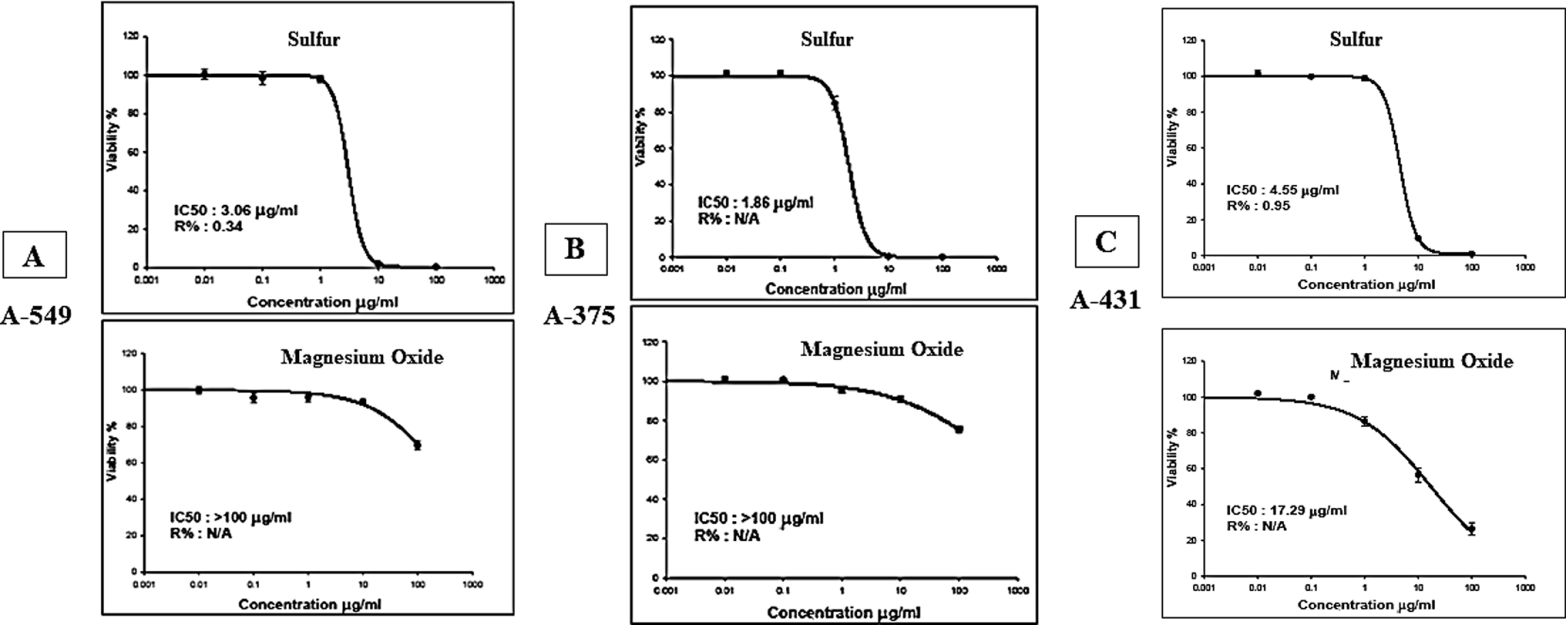
In-vitro SRB cytotoxicity assay (IC_50_) of Sulfur and Magnesium Oxide against (**A**): A-549 and (**B**): A-375, and (**C**): A-431 cell lines in increasing concentrations (0.01, 0.1, 1, 10, and 100 µg/mL). Data points are expressed as mean ± SD (*n* = 3).

The in vitro cytotoxic activity IC_50_ for Sulfur and MgO was assessed, and results are listed in Table S2 (supplementary table) and Fig. 12. The cytotoxicity was evaluated against three cancer cell lines, namely A-549, A-375, and A-431. The assays were conducted using SRB assay at 5 different concentrations 0.01, 0.1, 1, 10, and 100 µg/mL as the method described before.

From Fig. 12, it is truly clear that Sulfur displayed potent activity against all tested cancer cell lines, however, MgO showed potent activity on Human epidermoid skin carcinoma (skin/epidermis) (A-431) while activity was nearly negligible against the other two cell lines: A-549 and A-375.

#### Cell cycle arrest

**Fig. 13:**
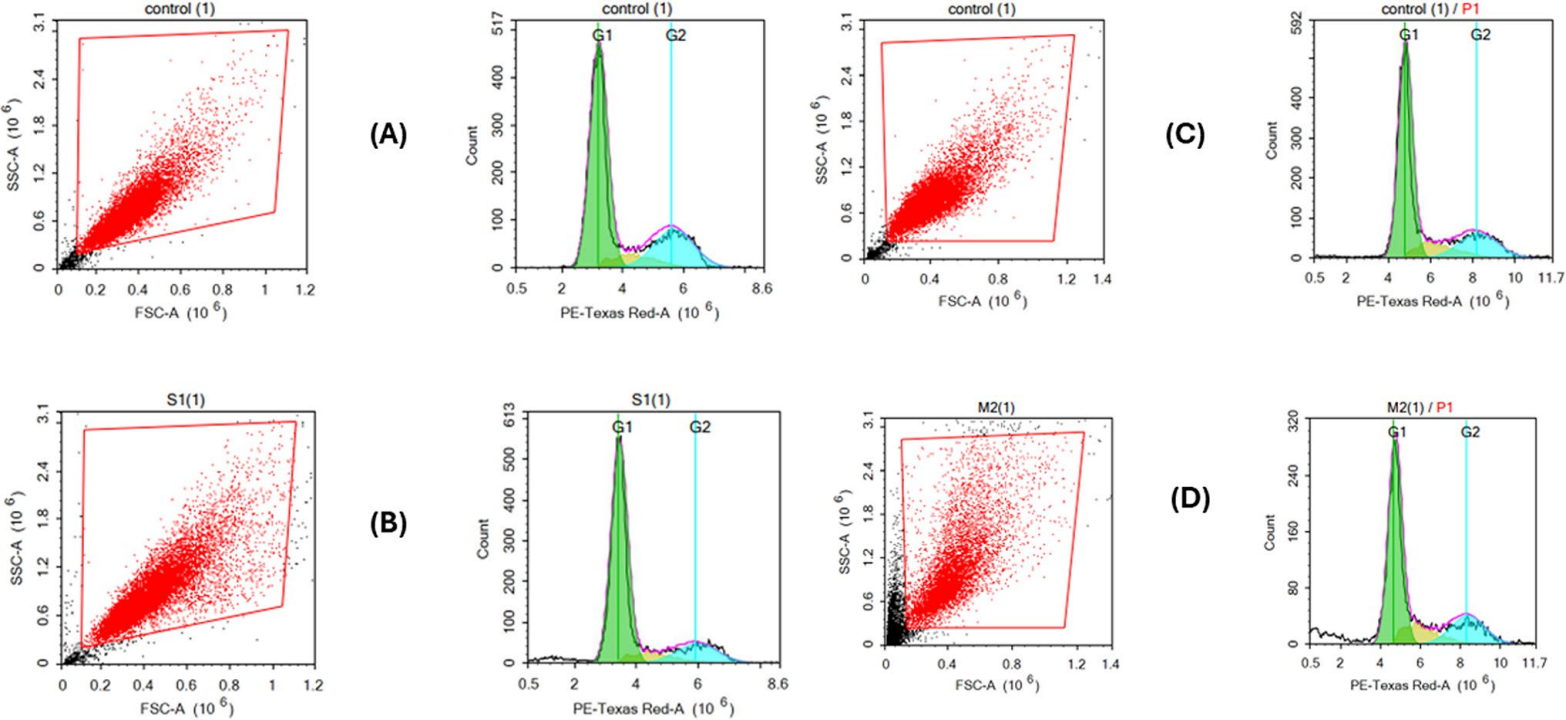
Effect of **(A)** Control (-ve), **(B)** Sulfur on the cell cycle distribution of A-375 cells, **(C)** Control (-ve), **(D)** Magnesium oxide on the cell cycle distribution of A-431 cells for 48 h and compared to control cells (**A** & **C**). Cell cycle distribution was determined using Flow cytometry analysis and different cell phases.

Further investigation for interference of Sulfur and Magnesium oxide to cell cycle phases of A-375 and A-431 cell lines respectively was performed as presented in Fig. 13.

Sulfur was found to significantly increase G0/G1-phase from 75.31 ± 1.75% to 80.93 ± 4.65% indicating its ant proliferative effect while it showed a slight increase in S-phase cell cycle arrest from 10.91 ± 0.16% to 10.18 ± 1.23%, a marked decrease in G2/M-phase from 29.34 ± 1.17% to 17.13 ± 2.57% and an increase in the SubG1-phase from 0.2 ± 0.14% to 4.01 ± 0.28%.

Magnesium induced S-phase arrest which significantly increased from 14.17 ± 0.09% to 18.49 ± 1.42%. Besides it increased G2/M-phase from 24.09 ± 2.21 to 28.8 ± 4.2 and SubG1-phase from 1.74 ± 0.34% to 8.07 ± 0.38% while it decreased G0/G1-phase from 73.77 ± 3.19% to 69.27 ± 1.52%.

#### Apoptosis/necrosis assessment using flow cytometry

**Fig. 14:**
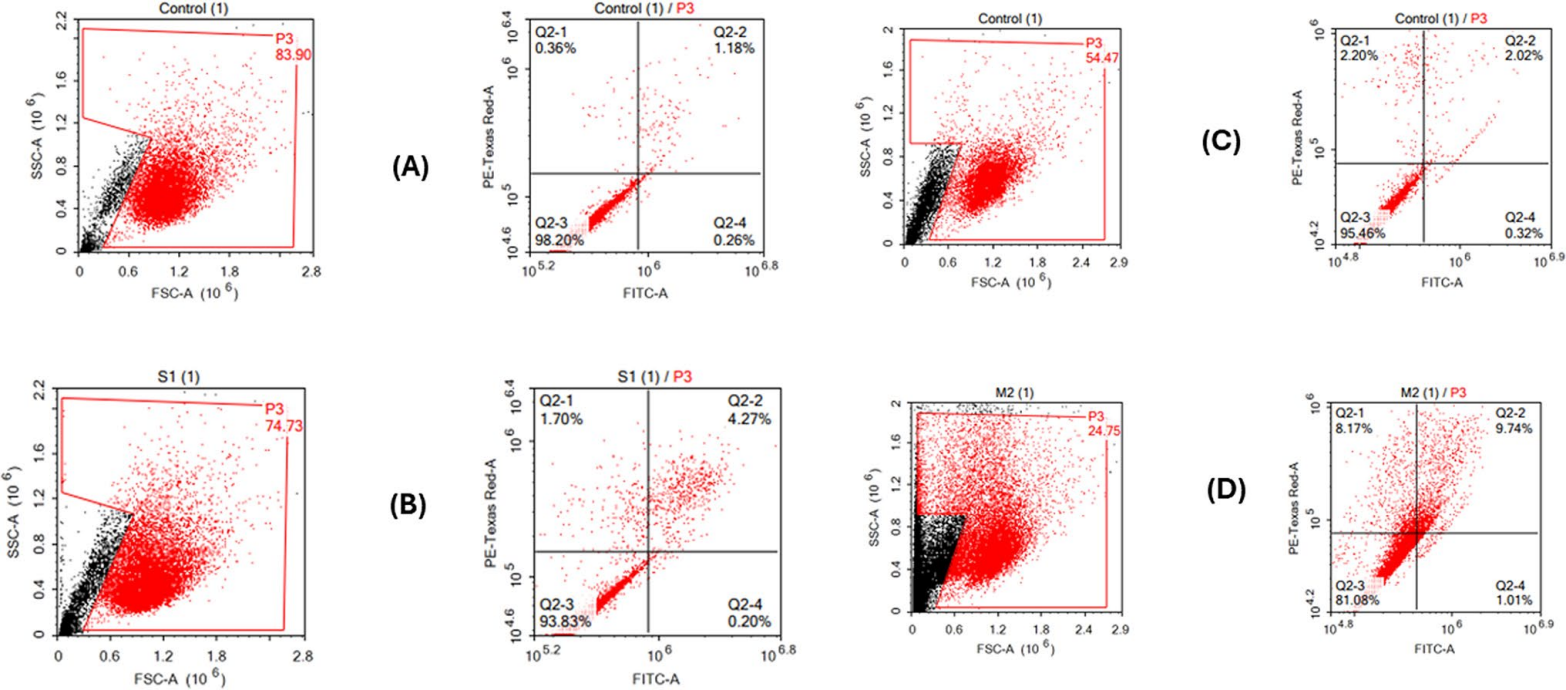
Mechanism of cell death induced by (**A**) Control (-ve), (**B**) Sulfur in A-375 cells, and (**C**) Control (-ve), (**D**) Magnesium oxide in A-431 for 48 h.

Annexin V-FITC/PI staining combined with flow cytometry was utilized to distinguish between the proportion of cells dying by necrosis and those undergoing apoptosis in both A-375 and A-431 cells. Based on the IC50 results, A-375 cells were treated with sulfur, while A-431 cells were treated with magnesium oxide for 24 and 48 h before conducting the apoptosis/necrosis differential assessment, as shown in Fig. 14.

In A-375 cells, sulfur significantly increased apoptosis by 1.5-fold in the early stage and 7-fold in the late stage compared to the control cells, leading to an overall increase in total cell death.

In A-431 cells, magnesium significantly increased apoptosis by 15.7-fold in the early stage and 8.7-fold in the late stage compared to control cells, resulting in an overall rise in total cell death. It is important to note that both sulfur and magnesium oxide also induced significant necrosis, indicating a non-specific killing effect.

In this section, DL experiments are done to test the performance of the given model to predict the in vitro cytotoxic activity IC_50_ for Vidarabine and its derivatives. Using the Python programming language, the experiments are conducted on a computer powered by an AMD Ryzen 7 processor clocked at 3 GHz, equipped with 16 GB of RAM, and running a 64-bit Windows 10 operating system. Several libraries and tools are used to develop DL models, including Numpy for numerical computations and array manipulation, Pandas for data handling and analysis, TensorFlow for DL model construction and training, Scikit-Learn for feature scaling, LIME for interpreting and explaining model predictions, and RDKit for generating molecular descriptors.

## Conclusion and future work

The cytotoxicity of Vidarabine Alkaloid (IC_50_, CC_50_, and SI) as a natural product was examined against lung cancer (A-549), human melanoma (A-375), human epidermoid skin carcinoma (skin/epidermis) (A-431) and two normal cell lines namely oral epithelial cell (OEC), human skin fibroblast (HSF). These activities were functionalized for the rediscovery of new compounds with promising anticancer activities. With the aid of DL, FRS, and XAI, Sulfur, and MgO were prioritized, selected, and examined against lung cancer (A-549), human melanoma (A-375), and Human epidermoid skin carcinoma (skin/epidermis) (A-431). Both Sulfur and MgO showed potent activity against A-431 cells while Vidarabine was inactive. Furthermore, Sulfur exhibited promising activities against A-549 and A-375 cell lines. Furthermore, flow cytometry and apoptosis studies for sulfur and magnesium oxide were performed. These findings open the gate for the implementation of Sulfur and MgO in preclinical and clinical phases especially for human epidermoid skin carcinoma. Additionally, Sulfur can be incorporated further for these previous drug discovery phases to treat lung cancer and human melanoma.

The proposed model in this paper represents a pioneering effort in the field of drug repurposing with AI, particularly in the context of Vidarabine alkaloid. Its ability to identify new features for compounds and its potential to enhance the field of drug discovery makes it a valuable contribution to both AI research and the quest for more efficient and impactful anticancer treatments. This work expresses the magnitude of the integration of AI with drug discovery. Therefore, in the future, the proposed model in this paper can be used as a pilot for further research for the rediscovery of new anticancer agents.

## Electronic supplementary material

Below is the link to the electronic supplementary material.

Supplementary Material 1

## Data Availability

The data is available at: https://github.com/dr-habeba/Exploring-Sulfur-and-Magnesium-Oxide-through-Integration-of-Deep-Learning-and-Fuzzy-RoughContact: heba.mamdouh@mu.edu.eg, or mamdouh.gomaa@mu.edu.eg.
