# Supplementary material for: Exploring the anticancer activities of Sulfur and magnesium oxide through integration of deep learning and fuzzy rough set analyses based on the features of Vidarabine alkaloid
Source: Sci Rep. 2025 Jan 17;15:2224. doi: 10.1038/s41598-024-82483-8 (PMC11742670; doi:10.1038/s41598-024-82483-8)
Supplement: Supplementary file 1 — Supplementary Material 1 [file 41598_2024_82483_MOESM1_ESM.pdf]

Table S1: SRB cytotoxicity assay showing Vidarabine IC_{50} on different cell lines in comparison to 5-FU as a reference drug.

Tested samples	IC_{50} (µg/ml)				
	Cancer Cell lines			Normal Cell lines	
	A-549	A-375	A-431	OEC	HSF
Vidarabines IC_{50}	6.97	25.78	> 100	9.47	18.31
5-FU IC_{50}	0.67	1.8	0.495	0.65	1.16

Table S2: SRB cytotoxicity assay showing Sulphur and MgO IC_{50} on different cell lines in comparison to 5-FU as a reference drug.

Tested samples	IC_{50}		
	Cancer Cell lines (µg/ml)		
	A-549	A-375	A-431
Sulfur	3.06	1.86	4.55
MgO	> 100	> 100	17.29
5-FU I	0.67	1.8	0.495

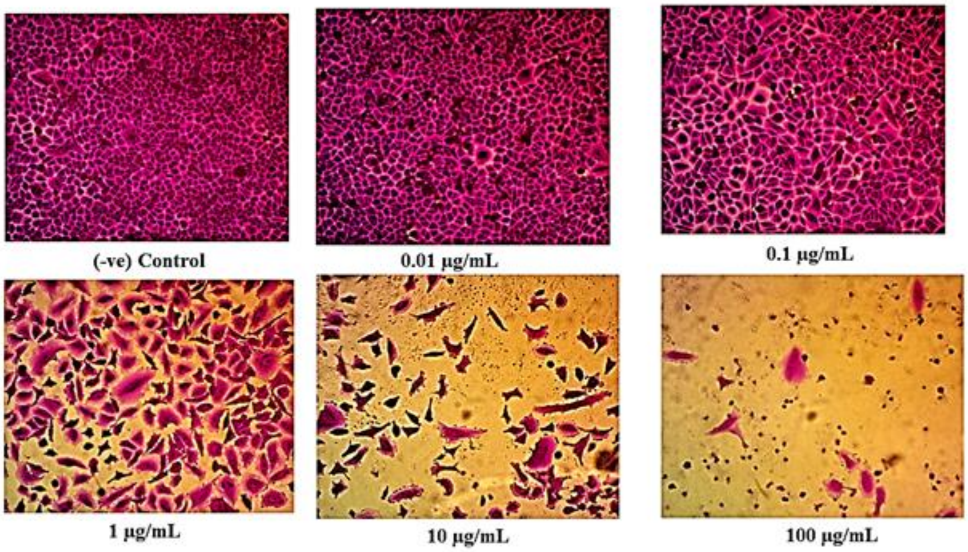

Fig. 7S. Optical microscope-stained images of SRB cytotoxicity assay (IC_{50}) of 5-FU (+ ve control) against A-549 cell line at 5 different concentrations (0.01, 0.1, 1, 10 and 100 µg/mL).

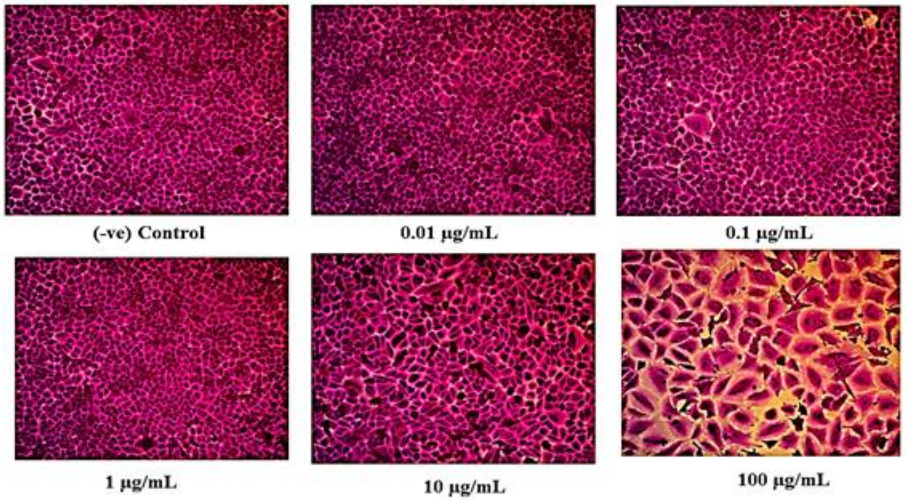

Fig. 8S. Optical microscope-stained images of SRB cytotoxicity assay (IC_{50}) of Vidarabine against A-549 cell line at 5 different concentrations (0.01, 0.1, 1, 10 and 100 µg/mL).
